# Time-dependent effects of imatinib in human leukaemia cells: a kinetic NMR-profiling study

**DOI:** 10.1038/sj.bjc.6604946

**Published:** 2009-03-03

**Authors:** J Klawitter, N Anderson, J Klawitter, U Christians, D Leibfritz, S G Eckhardt, N J Serkova

**Affiliations:** 1 Department of Anesthesiology, University of Colorado, Denver, CO 80045, USA; 2 Department of Chemistry, University of Bremen, Bremen 28334, Germany; 3 Center of Drug Research and Medical Biotechnology, Fraunhofer Institute for Toxicology and Experimental Medicine, Hannover 30625, Germany; 4 Developmental Therapeutics and GI Malignancies Program, Division of Medical Oncology, Department of Medicine, University of Colorado, Denver, CO 80045, USA; 5 University of Colorado Cancer Center, University of Colorado, Denver, CO 80045, USA

**Keywords:** imatinib, apoptosis, metabonomics, nuclear magnetic resonance

## Abstract

The goal of this study was to evaluate the time course of metabolic changes in leukaemia cells treated with the Bcr-Abl tyrosine kinase inhibitor imatinib. Human Bcr-Abl^+^ K562 cells were incubated with imatinib in a dose-escalating manner (starting at 0.1 *μ*M with a weekly increase of 0.1 *μ*M imatinib) for up to 5 weeks. Nuclear magnetic resonance spectroscopy and liquid-chromatography mass spectrometry were performed to assess a global metabolic profile, including glucose metabolism, energy state, lipid metabolism and drug uptake, after incubation with imatinib. Initially, imatinib treatment completely inhibited the activity of Bcr-Abl tyrosine kinase, followed by the inhibition of cell glycolytic activity and glucose uptake. This was accompanied by the increased mitochondrial activity and energy production. With escalating imatinib doses, the process of cell death rapidly progressed. Phosphocreatine and NAD^+^ concentrations began to decrease, and mitochondrial activity, as well as the glycolysis rate, was further reduced. Subsequently, the synthesis of lipids as necessary membrane precursors for apoptotic bodies was accelerated. The concentrations of the Kennedy pathway intermediates, phosphocholine and phosphatidylcholine, were reduced. After 4 weeks of exposure to imatinib, the secondary necrosis associated with decrease in the mitochondrial and glycolytic activity occurred and was followed by a shutdown of energy production and cell death. In conclusion, monitoring of metabolic changes in cells exposed to novel signal transduction modulators supplements molecular findings and provides further mechanistic insights into longitudinal changes of the mitochondrial and glycolytic pathways of oncogenesis.

Chronic myeloid leukaemia (CML) is a clonal myeloproliferative disorder that is characterised by excessive production and premature release of myeloid cells. The molecular hallmark of CML is the Philadelphia (Ph) chromosome that results from the t(9;22) q(34;11) reciprocal chromosomal translocation. The Ph chromosome contains the *BCR-ABL* hybrid gene (Nowell and Hungerford, 1960; Rowley, 1973) that encodes an oncogenic fusion protein, Bcr-Abl, of 190, 210 and 230 kDa (Heisterkamp *et al*, 1983). The fusion proteins possess a constitutively active cytoplasmic Abl tyrosine kinase domain that does not block differentiation, but enhances the proliferation and viability of myeloid cells. Furthermore, Bcr-Abl tyrosine kinase protects haematopoietic progenitor cells from spontaneous apoptosis as well as from that induced, for example, by chemotherapeutic drugs (Bedi *et al*, 1995). Although the precise mechanism by which Bcr-Abl exercises its antiapoptotic properties is unknown, several possible explanations have been reported, including increased expression of the antiapoptotic protein, Bcl-xL (Amarante-Mendes *et al*, 1998) and induction of the nuclear factor-*κ*B (Reuther *et al*, 1998).

Targeting the tyrosine kinase activity of Bcr-Abl is an attractive therapeutic strategy in CML. Several tyrosine kinase inhibitors, such as imatinib mesylate (CGP57148B or Gleevec, Novartis Pharma, Basel, Switzerland), have been developed. Imatinib competitively inhibits the binding of ATP to the kinase domain of the Abl at low micromolar concentrations (Druker *et al*, 1996; Buchdunger *et al*, 2000). Its binding abrogates the activity of the Bcr-Abl oncoprotein through the inhibition of Bcr-Abl autophosphorylation and substrate phosphorylation leading to the inhibition of cell proliferation and induction of apoptosis (Druker *et al*, 1996; Deininger *et al*, 1997; Gambacorti-Passerini *et al*, 1997).

Nuclear magnetic resonance (NMR) spectroscopy has successfully been used as a tool to evaluate the efficacy of anticancer therapies on the basis of the altered metabolic oncogenesis network (Evelhoch *et al*, 2000; Gillies *et al*, 2000). It is a technique that yields metabolic and bioenergetics information from cells or tissues in a dynamic manner, and can be applied non-invasively *in vivo*. For example, variations in the rate of cell proliferation, cell-cycle progression and apoptosis cause changes in glucose cell metabolism and energy state (Evelhoch *et al*, 2000; Gillies *et al*, 2000; Griffin and Shockcor, 2004). Moreover, the metabolic response to anticancer treatment resulting in apoptosis and necrosis through the specific inhibition of a signal transduction pathway often occurs before morphological or histological changes are evident (Shih *et al*, 2005). Thus, a large number of reported metabolic changes involved in apoptotic processes, such as an increased concentration of polyunsaturated fatty acids (PUFAs; Blankenberg *et al*, 1996; Robertson and Orrenius, 2000; Griffin *et al*, 2003) and triacylglycerol (TAG; Blankenberg *et al*, 1997; Al-Saffar *et al*, 2002), as well as changes in choline-containing metabolites from the Kennedy pathway, such as choline, phosphocholine (PC) and glycero-PC (GPC; Glunde *et al*, 2002; Valonen *et al*, 2005), can easily be observed by ^1^H- and ^31^P-NMR.

In this study, we investigated the early and late biochemical and molecular events taking place during imatinib-induced apoptosis in human leukaemia K562 cells. Metabolic ^1^H-, ^13^C- and ^31^P-NMR were used for detection of changes in oncogenesis-related metabolic pathways, such as phospholipid, glucose and energy metabolism. The changes in metabolite patterns were correlated with the changes in protein expression and intracellular imatinib concentrations.

## Materials and methods

### Cell culture and imatinib treatment

The human Bcr-Abl^+^ leukaemia cell line, K562, was purchased from the German Collection of Microorganisms and Cell Cultures (DSMZ, Braunschweig, Germany). The cells were grown in an RPMI 1640 culture medium containing 10% foetal bovine serum (FBS) and were kept at 37°C with 95% air/5% CO_2_. Imatinib was kindly provided by Dr Buchdunger (Novartis Pharma, Basel, Switzerland). The K562 cells were treated with increasing concentrations of imatinib (increase of 0.1 *μ*M per week), whereas the control cells were kept under the same conditions without the addition of imatinib. The medium containing fresh imatinib was changed at the beginning of the new treatment period and at the beginning of every feeding/subcultivation cycle. The dose escalation of 0.1 *μ*M imatinib per week has been chosen on the basis of previously published protocols (Mahon *et al*, 2000; Melo and Chuah, 2007), in which leukaemia cells, such as K562, were exposed to increasing concentrations of imatinib to induce resistance against 1 *μ*M imatinib. The increments of imatinib increase were kept low at 0.1 *μ*M per week to ensure that the cells were able to adapt to the changing conditions while still exhibiting metabolic response to drug exposure. Cell extracts, lipids and media fractions were collected and analysed by NMR after 96 h, 1 (0.1 *μ*M), 2 (0.2 *μ*M) and 4 weeks (0.4 *μ*M imatinib).

Cell proliferation and viability were examined by cell counting using the trypan blue exclusion method.

### Western blot assay of phospho-tyrosine kinase expression

Phospho-tyrosine kinase activity was assessed by western blot analysis. Protein lysates were analysed by electrophoresis on 7–14% SDS–PAGE gels, and blotted onto polyvinylidene difluoride membranes by semidry electrophoretic transfer. The gel was then stained with Coomassie Blue. The membrane was blocked with 3% dry milk and 0.1% Tween 20 in PBS and incubated with primary antibodies (4G10 antiphosphotyrosine and *β*-actin, Santa Cruz Biotechnology, Santa Cruz, CA, USA). The filters were washed and incubated with horseradish peroxidase-conjugated donkey antirabbit antibody, and quantities of proteins relative to *β*-actin were determined by densiometry.

### Flow cytometry analysis on apoptosis Abl and p-glycoprotein

To compare protein expression levels, the cells were washed, fixed, permeabilised and stained with the following antibodies: anti-Abl (Cell Signaling Technology Inc., Beverly, MA, USA), anti-p-glycoprotein (MRK16, Alexis Biochemicals, San Diego, CA, USA) and anti-Glut-1 (Dako Cytomation, Carpinteria, CA, USA). The mean fluorescence intensity (*x*-mean all) of control and treated cells was used for the quantification of protein expression. For measurements of apoptosis and necrosis, the cells were incubated with a YoPro-1/PI dye (Molecular Probes, Eugene, OR, USA). The analysis was performed by using either a Becton Dickinson FACSCalibur (Franklin Lakes, NJ, USA) or by using a Beckman Coulter FC500 (Brea, CA, USA) instrument at the Flow Cytometry Cancer Core Facility, University of Colorado, Denver.

### Cell extraction for NMR spectroscopy

For NMR experiments, the cells (∼10^8^) were incubated with 5 mmol l^−1^ [1-^13^C]glucose (Cambridge Isotope Laboratories, Andover, MA, USA) for the last 4 h of treatment before perchloric acid (PCA) extraction. All cell extractions were performed using a previously published PCA extraction protocol (Gottschalk *et al*, 2004). Lyophilised PCA cell extracts were redissolved in 0.6 ml and media in 1 ml of deuterium oxide. After centrifugation, the supernatants were neutralised to pH 7.2 to allow for precise chemical shift assignments. Lipid extracts were redissolved in 1 ml mixture of deuterated methanol/chloroform (2 : 1, v/v).

### NMR spectroscopy experiments

All high-resolution ^1^H- and ^13^C-NMR experiments were performed using a Bruker 500 MHz DRX spectrometer equipped with an inverse 5-mm TXI probe (Bruker BioSpin, Fremont, CA, USA). All ^31^P-NMR experiments were performed using a 300 MHz Bruker Avance system with a 5-mm QNP probe. For proton NMR, a standard water presaturation pulse programme was used for water suppression; spectra were obtained at 12 p.p.m. spectral width, 32K data arrays and 64 scans with 90° pulses applied every 15.2 s to allow for full relaxation of all protons. Trimethylsilyl propionic-2,2,3,3,-d_4_ acid (TMSP; 0.5 mmol l^−1^ for cell PCA extracts and 1.1 mmol l^−1^ for media and lipids) was set to 0 p.p.m. for chemical shift reference and used as an external standard for metabolite quantification. ^13^C-NMR spectra with proton decoupling were recorded using the C3-lactate peak at 21 p.p.m. as chemical shift reference (spectral width was 150 p.p.m., 16K data arrays and 20K scans applied every 3 s). For absolute quantification of ^13^C metabolites, calculations according to Zwingmann and Leibfritz, (2003) were made.

^31^P-NMR spectra were obtained using the spectral width of 50 p.p.m. and 16K data arrays, 6–10K scans applied every 3.5 s. Before ^31^P-NMR spectra were recorded, EDTA (100 mmol l^−1^) was added to each PCA extract to complex divalent cations, and the pH was adjusted to 7.2. Methylene diphosphonic acid (2 mmol l^−1^) in a sealed capillary was used as chemical shift reference (18.6 p.p.m.), and as an external standard for metabolite quantification using ^31^P-NMR. All data were processed using the Bruker 1D-WINNMR Software (Bruker Biospin).

To verify the metabolite assignments in extracts, two-dimensional (2D)-H,C-HSQC (heteronuclear single quantum correlation) was carried out. All 2D-measurements were performed using a 600 MHz Bruker DRX spectrometer system (Bruker, Karlsruhe, Germany). The experiments were acquired with 512 t1-increments and 32 scans per increment. All 1D-NMR experiments were performed at the Metabolomics NMR University of Colorado Cancer Center Core.

### HPLC/MS analysis

For the nucleotide concentration measurements (ATP, ADP, NAD^+^, AMP, UTP and CTP), an Agilent 1100LC system coupled to an ESI/MSD detector was used. The cells (10^8^ cells each) were extracted using PCA (see above), and the high-performance liquid chromatography/mass spectrometry (HPLC/MS) method was validated (Klawitter *et al*, 2007). The assay was linear from a 10 to 1000 pmol nucleotide amount on the column using *N*6-(aminohexyl)-adenosine-5′3′-diphosphate (Sigma-Aldrich, St Louis, MO, USA) as an internal standard. The cell extracts were diluted 1 : 5, so that the measured concentrations fell within the linear range of quantification.

Intra- and extracellular imatinib concentrations were determined using a validated LC/MS/MS assay. Briefly, after ultrasonic treatment and protein precipitation with methanol/60 mM zinc sulphate (80/20 v/v), the cell samples (each 10^7^ cells) were extracted on-line. After activation of the column switching-valve, the extracts were back-flushed onto the analytical column (Luna C18, 50 × 4.6 mm, 5 *μ*m, Phenomenex, Torrance, CA, USA). The HPLC system, interfaced with a triple quadrupole MS (API4000, Applied Biosystems, Foster City, CA, USA), was run in positive multiple reaction monitoring (MRM) mode. Peak area ratios obtained from the MRM mode of the mass transition for imatinib (*m*/*z* 494 → 394) and from the internal standard trazadone (Sigma-Aldrich; *m*/*z* 372 → 176) were used for quantification. The lower limit of quantification was 0.03 ng ml^−1^ cell homogenate from 10^7^ cells, and the assay was linear between 0.03 and 75 ng ml^−1^. Interday accuracy was between 90–110% of the nominal concentrations, and interday precision was always equal to or better than 10%. No significant ion suppression or other matrix interference was detected. Samples were stable in the autosampler at +4°C for at least 24 h, and the drug was stable in cell homogenates for at least three freeze-thaw cycles. All HPLC/MS analyses were performed at the Clinical Nutrition Research Unit Mass Spectrometry Core (University of Colorado, Denver).

### Statistical analysis

The number of observations was *n*=3 or higher for all experiments. All numerical data are presented as mean±s.d. One-way analysis of variance (Estanyol *et al*, 1999) was used to determine differences among groups (untreated *vs* time-course treated). The significance level was set at *P*<0.05 for all tests (SigmaPlot-version 9.01, Systat Software, Point Richmond, CA and SPSS version 14.0, SPSS Inc., Chicago, IL, USA).

## Results

### Cell proliferation and cell death

Following established protocols (Mahon *et al*, 2000; Melo and Chuah, 2007), K562 cells were exposed to increasing concentrations of imatinib to induce resistance against 1 *μ*M imatinib. Exposure of K562 cells to 0.1 *μ*M imatinib for 96 h led to a significant change neither in cell proliferation nor in cell cycle distribution. The time period between the second (0.2 *μ*M imatinib) and third week of treatment (0.3 *μ*M imatinib) was marked by a decreased cell proliferation rate (−34% decrease in treated *vs* untreated cells). Finally, 4 weeks of treatment with imatinib reduced the number of viable cells to −46%, and the prolonged incubation to 0.5 *μ*M imatinib (5 weeks) reduced it further by 70%.

**Figure 1:**
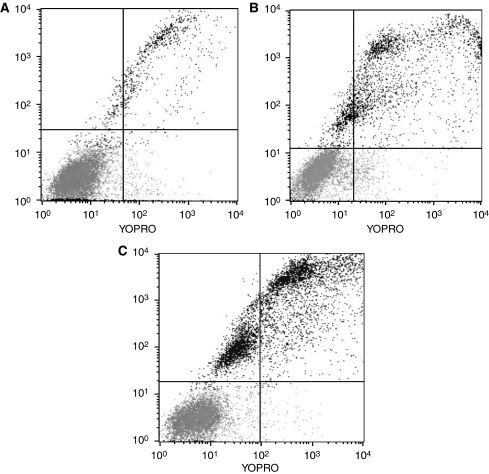
Flow cytometry plots of K562 cells: (**A**) untreated cells (control); (**B**) treatment with 0.1 *μ*M imatinib for 1 week; and (**C**) treatment with increasing imatinib concentrations for 4 weeks (0.1 *μ*M imatinib increase per week; end concentration 0.4 *μ*M). In each plot, four quadrants with different cell viabilities were distinguished: bottom left, living cells; upper left, necrotic cells; bottom right, early apoptotic; upper right, apoptotic cells. In all cells, 3–8% necrotic cells were observed because of the staining procedures.

**Figure 2:**
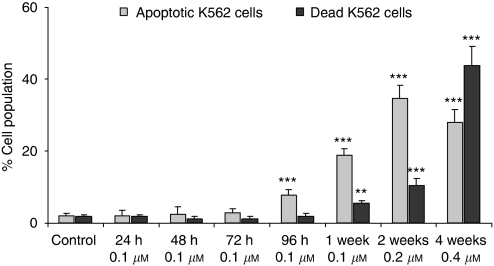
Time-dependent and imatinib-concentration-dependent number of apoptotic and necrotic cells in K562 cells. The data are presented as means+s.d. of three independent flow cytometry experiments and are given as % of cell population (set at 100%); significance levels: ^*^P<0.05; ^**^P<0.005; ^***^P<0.001.

After staining with YoPro and PI, the living cells (Figure 1A–C, bottom left corner) were non-fluorescent as assessed by flow cytometry, although having the ability to exclude both dyes. Early apoptotic cells that were labelled with YoPro, but still maintaining the ability to exclude PI, were located in the bottom right corner. Finally, apoptotic (upper right corner) and necrotic (upper left corner) cells were both YoPro and PI^+^. If untreated, most of the K562 cells were viable cells (bottom left corner) without significant signs of apoptosis as can be seen on flow cytometry plots (Figure 1A). After 96 h of treatment with 0.1 *μ*M imatinib, apoptosis was initiated and widespread with increasing incubation time and dose (weekly increase by 0.1 *μ*M imatinib; Figures 1B and 2). After 4 weeks (0.4 *μ*M imatinib), 44% apoptotic cells were detected in K562 cells (Figures 1C and 2).

Another method used for measurements of cell viability and membrane stability was staining with Trypan blue. No increase in the number of stained, dead cells occurred until 2 weeks. At this time point, approximately 20% total dead cells were observed. This confirmed the flow cytometry findings (Figure 2). Thereafter, the number of dead cells increased exponentially. As aforementioned, after 4 weeks, more then 46% of cells were dead.

### Changes in Bcr-Abl protein expression and protein phosphorylation

**Figure 3:**
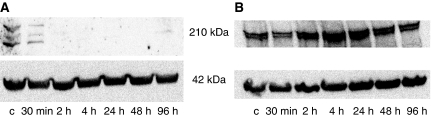
Western blot (**A**) of p-tyr expression in K562-treated cells in the first 96 h of imatinib treatment (0.1 *μ*M). Changes in c-Abl protein expression were also assessed by western blotting (**B**). All western blots were carried out in triplicate.

Incubation with imatinib significantly reduced the fluorescence intensity of the p-tyr staining in K562 leukaemia cells. Western blot analysis showed that the p-tyr band (at 210 kDa) completely disappeared after 2 h of exposure to 0.1 *μ*M imatinib (Figure 3A). Flow cytometry revealed a significant decrease to less than 30% of the expression of controls after 96 h of incubation (*P*<0.0001, *n*=3). Moreover, the level of the c-Abl protein was almost three-fold higher than that in the controls after 24–48 h (likely trying to compensate for decreased tyrosine kinase activity), but decreased to control values after 96 h of treatment (Figure 3B).

### Glucose metabolism after imatinib treatment

After 1 week of 0.1 *μ*M imatinib treatment, glucose metabolism in K562 cells shifted from predominantly cytosolic glycolysis, as indicated by decreased lactate production, towards the mitochondrial Krebs cycle, as indicated by elevated glutamine and glutamate synthesis. As calculated from the ^13^C-NMR spectra of cell extracts, the concentration of *de novo* produced [3-^13^C]-lactate decreased to −39% (*P*<0.05), indicating an inhibition of cytosolic glycolytic activity. In parallel, mitochondrial Krebs cycle-derived metabolite production increased by +44% (*P*<0.05, *n*=5; Table 1A). Further treatment of K562 cells (2 weeks with 0.2 *μ*M imatinib) almost completely inhibited glycolysis (^13^C-lactate concentration diminished to −85% from control, *P*<0.005, all above *n*=5) and Krebs cycle activity started to decline (Table 1A), reaching its minimum of −54% from baseline (*P*<0.005, *n*=3) after 4 weeks (imatinib at 0.4 *μ*M; Table 1A). The inhibition of glycolytic and later mitochondrial glucose metabolism resulted in intracellular glucose accumulation, despite imatinib-mediated decrease in glucose uptake from the media (see below).

### Glucose uptake/lactate release

The time course of imatinib effects on glucose uptake and lactate release was studied in the media of K562 cells treated with imatinib (Table 1B). In treated cells, lactate export was already reduced 24 h after addition of imatinib, followed by decreased glucose uptake after 48 h (Table 1B). Impaired glycolytic activity of leukaemia cells during treatment with imatinib led to extracellular and intracellular glucose accumulation with a subsequent loss of lactate export into the extracellular milieu of K562 cells (Table 1B). This was the earliest metabolic effect observed in imatinib-treated cells that started at 24 h and reached its plateau after 96 h of treatment (see Table 1B).

### Energy state after imatinib treatment

**Figure 4:**
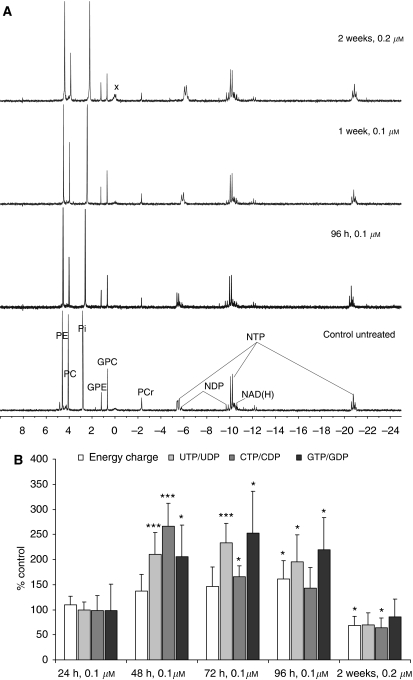
(**A**) Representative ^31^P-NMR spectra of K562 cell extracts. Cells were incubated with 0.1 *μ*M imatinib for 96 h or 1 week and with 0.2 *μ*M imatinib for 2 weeks, and compared with untreated controls. (**B**) Concentration ratios of high-energy phosphates as calculated from the HPLC/MS data of K562 cell extracts. Energy charge was calculated as ((ATP+0.5^*^ADP)/(AMP+ADP+ATP)). The values were calculated per number of cells ( × 10^7^) and are given as % of controls set to 100% and represent means+s.d. of three independent experiments. Significance levels: ^*^P<0.05; ^**^P<0.005; ^***^P<0.001. Peak assignment: PC=phosphorylcholine; PE=phosphorylethanolamine, Pi=inorganic phosphate; GPC=glyceryl-PC; GPE=glycerylphosphoethanolamine; X=other phosphodiesters; PCr:=phosphocreatine; NAD(H)=nicotine amide adenine dinucleotide; NTP=nucleotide triphosphates; NDP=nucleotide diphosphates. The chemical shift assignment was based on the methylene diphosphonic acid (MDP) signal at 18.6 p.p.m.

The increase in mitochondrial glucose metabolism during the first week of treatment was accompanied by a higher energy state of Bcr-Abl^+^ K562 cells as indicated by ^31^P-NMR spectra of cell extracts (Figure 4A). The cell energy balance (calculated as nucleoside triphosphate to nucleoside diphosphate (NTP/NDP) ratio) increased from 6.7 in control to 9.4 after 1 week of imatinib treatment (*P*<0.05, *n*=4, Table 1A). From that point on, the energy balance declined to control values after 2 weeks and reached −74% of the control values after 4 weeks of treatment (Table 1A). This went parallel with the activation of the apoptosis cascade. At the same time, a noticeable decrease in phosphocreatine concentration was observed and it reached its minimal value again after 4 weeks of treatment (the signal was not detectable, Table 1A).

As the magnetic field employed for our ^31^P-NMR experiments could not resolve specific trinucleotides, additional HPLC/MS analyses were performed to determine concentrations of specific nucleotides. The results are presented in Figure 4B. These analyses confirmed the ^31^P-NMR results. Imatinib-treated cells accumulated high-energy phosphates during the first week of treatment. Thereafter, the tri-/dinucleotides ratio decreased and remained low after 2 weeks of treatment.

High-performance liquid chromatography/mass spectrometry analyses of NAD^+^ concentrations showed a significant decrease after just 96 h of incubation in imatinib-treated cell lines and an even further reduction with increasing treatment duration and drug concentration (which can be explained by decreased glycolytic activity). After 2 weeks of imatinib treatment, the intracellular NAD^+^ concentration decreased from 1.3 to 0.5 *μ*mol l^−1^ per 10^7^ cells and remained at this low level up to 4 weeks of treatment.

### Changes in phospholipid metabolism

Another significant difference in the metabolic response of imatinib-treated K562 cells compared with controls was a decrease of PC and phosphatidylcholine (PtdCho). Phosphocholine (the precursor of PtdCho, the major phospholipid in the cell membrane) decreased by −25% in the first week (*P*<0.05, *n*=5) and further by −47% in the following week of treatment with imatinib (*P*<0.001, *n*=4; Table 1A), as calculated from the ^31^P-NMR spectra of PCA cell extracts (Figure 4A). The PC concentration of about 50% of controls remained at the same level in the following 2 weeks of treatment. The decrease in PC resulted in overall declined ratios of phosphomonoester (PME) to phosphodiester (Pozzi *et al*, 2004). These are surrogate markers for phospholipid-pool turnover (−38 and −52% of control after 2 and 4 weeks, respectively, *P*<0.05).

Changes in PtdCho concentration, the major phospholipid in the cell membrane, reflected the same profiles as measured by ^1^H-NMR of lipid fractions. The PtdCho concentration decreased by −13% of control (although not significant) after the first week and continued to decrease by −24% (*P*<0.05) after 2 weeks, with a minimum of −50% of control observed after 4 weeks of treatment (*P*<0.005, all *n*=3).

### Changes in neutral lipids and fatty acids

With increasing apoptosis rate, increased signal intensity of PUFAs was observed after 2 and 4 weeks of treatment (+50 and +81% from control, respectively, *P*<0.05). In addition, the concentration of methylene/methyl (CH_2_/CH_3_) resonances of fatty-acid chains increased as well (CH_2_: +38 and +72% of control after 2 and 4 weeks, both *P*<0.001 with *n*=3 and CH_3_: +20% (not significant) and +35% (*P*<0.05) of control after 2 and 4 weeks, respectively, *n*=3). *De novo* diacylglycerol (DAG) and TAG syntheses were not studied, but the integration of the TAG signal in ^1^H-MRS spectra of lipids revealed that its total concentration increased by +21 and +81% of controls following 2 and 4 weeks of imatinib treatment (*n*=3, *P*<0.05, respectively).

### Intra- and extracellular imatinib concentrations

Intracellular imatinib concentrations did not significantly change between 24 and 72 h of treatment. After 96 h, accumulation of imatinib was seen in K562-treated cells (Table 2). After the first week, the cell media was changed and fresh 0.2 *μ*M imatinib was added. The total imatinib concentration was again measured at the end of 2 weeks (calculated as the sum of intra- and extracellular concentrations), and showed that 20% of imatinib had degraded or had been metabolised after this period of time. The same procedure was repeated after 3 and 4 weeks. At the end of 4 weeks, the concentration of imatinib was 13% less than that of the amount added.

### Expression of p-glycoprotein

Assessment of the effects of incubation time and imatinib concentrations showed that K562 cells were not subject to any changes in p-glycoprotein expression.

## Discussion

The discovery and development of imatinib has shown that it is possible to produce rationally designed, molecular-targeted drugs for treatment of a specific cancer. Such designed compounds can affect not only one, but several targets, allowing for therapeutic efficacy in several diseases. One of the major concerns with imatinib treatment is the development of drug resistance. Earlier research and clinical reports have indicated that the changes in glucose metabolism may be related to, or even be predictive of, the development of imatinib resistance (Serkova and Boros, 2005).

**Figure 5:**
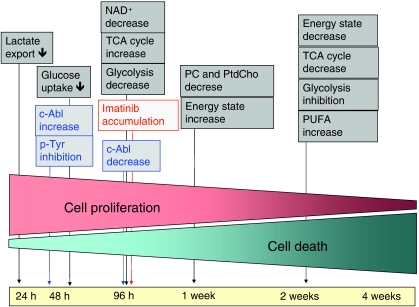
Summary of time-dependent effects of imatinib on metabolism of human Bcr-Abl^+^ K562 cells. NAD^+^=nicotinamide adenine dinucleotide; PC=phosphocholine; PtdCho=phosphatidylcholine; PUFA=polyunsaturated fatty acids; TCA=tricarboxylic acid.

In this study, cellular metabolic responses to imatinib treatment, including mitochondrial activity and glucose utilisation, energy production and membrane turnover were investigated in a time-dependent matter following the cells on their path in becoming resistant to imatinib (for a summary of these effects, please refer to Figure 5). The imatinib concentration chosen (0.1 *μ*M) had successfully been used in earlier studies (Gottschalk *et al*, 2004). This concentration was confirmed not to cause cytotoxic effects during short-term administration (up to 96 h). However, long-term treatment (from 1 to 4 weeks) with a dose-escalation (0.1 *μ*M imatinib per week) caused significantly higher apoptosis rates, and after 4 weeks, 46% of the treated cells were dead. The exact time course for imatinib-mediated metabolic changes is presented in Figure 5, with initial glycolysis inhibition, followed by decrease in glucose uptake, changes in mitochondrial TCA cycle derivatives and energy production. Secondary changes were related to membrane composition as seen from lipid and phospholipid turnover (Henke *et al*, 1998).

Tumour cells exhibit elevated glycolysis rates even in the presence of oxygen, the so-called Warburg effect (Warburg, 1956). Increased expression of the glucose transporter, Glut-1, and increased hexokinase activity seem to be associated with the acceleration of glycolysis in tumours (Asano *et al*, 2000). Studies in gastrointestinal stromal tumour patients have shown a rapid decrease in fluorinated deoxyglucose (FDG) uptake, sometimes as early as 24 h after administration of imatinib (Van den Abbeele and Badawi, 2002). Earlier studies have also reported that imatinib regulates glucose flux through downregulation and translocation of Glut-1 transporters from the cell membrane into the cytoplasm in human leukaemia cells (Boren *et al*, 2001).

When treated with imatinib, leukaemia cells showed complete and rapid inhibition of their tyrosine kinase activity (2 h), followed immediately by decreased glycolysis rates (24 h). This is consistent with the reversal of the Warburg effect. The suppression of glycolysis resulted in decreased [1-^13^C]glucose uptake from the incubation media at 48 h. This supported earlier findings (Gottschalk *et al*, 2004). Although the [3-^13^C]-lactate concentration decreased, a simultaneous increase in C4-glutamate production occurred during the first week of the observation period. Production of high-energy phosphates (NTP) was elevated most likely because of increased mitochondrial energy production. These observations and the suggestion that imatinib stimulates mitochondrial metabolism and reduces anaerobic glycolysis in Bcr-Abl^+^ leukaemia cells in the first 96 h of treatment confirmed the results reported by Gottschalk *et al* (2004).

When cells are exposed to cytostatic agents, there are two major types of cell death: apoptosis and necrosis. Cell shrinkage, DNA damage, chromatin condensation and blebbing of the plasma and alteration of plasma membrane phospholipid organisation with phosphatidylserine externalisation are major characteristics of apoptosis (Williams, 1991; Wood and Youle, 1994). Necrosis is generally characterised by swelling of cells and mitochondria, scattered chromatin condensation and loss of plasma membrane integrity because of an overwhelming physical cell injury (Williams, 1991; Nicotera and Leist, 1997).

In the time period between 96 h and 2 weeks of treatment, the antiproliferative and proapoptotic properties of imatinib were revealed in our cell model. After the first week of treatment, decreased cell proliferation rate and increased number of apoptotic cells became detectable and were enhanced with longer incubation times and higher imatinib concentrations. Short-term incubation with 0.1 *μ*M imatinib led to an increase in mitochondrial activity and energy balance and/or charge. The maxima were reached after 1 week. Thereafter, both mitochondrial activities and energy charge started to decrease. After 96 h, the first signs of apoptosis were observed, the cells were highly active and have maintained their elevated mitochondrial activity. This is consistent with the literature, in which it is known that the apoptotic process demands high energy for its development (Eguchi *et al*, 1997; Zamaraeva *et al*, 2005).

Recent studies showed that cells undergoing apoptosis show the same or even better energy status as long as the cells maintained a highly active metabolism (such as various biosynthesis processes including lipid biosynthesis, DNA fragmentation and so on; Budd *et al*, 2000; Zamaraeva *et al*, 2005). As apoptosis progresses, energy metabolism declines rapidly. In our case with increasing number of apoptotic and dead cells, a marked decrease in PCr was observed with a subsequent decrease in NTP concentrations. Similar observations were made by Henke (1996), whose investigations on miltefosine-induced apoptosis revealed a ‘stable’ NTP state as long as the cells maintained a highly active metabolism. In addition to the energy state, decreased NAD^+^ concentration may also serve as early indicators for ongoing imatinib-induced apoptosis. Earlier studies have shown that other antitumour agents initiate the apoptotic cascade by decreasing NAD^+^ concentrations (Wosikowski *et al*, 2002).

Phosphocholine, a major precursor for membrane synthesis, is known to increase in rapidly proliferating cancer cells, including leukaemia cells (Franks *et al*, 2002; Ackerstaff *et al*, 2003). Its reduction was significant after the first week and even enhanced after 2–4 weeks of imatinib treatment. The reduction in PC is within the response of other cell types and tumours to treatment as described earlier (Glunde and Serkova, 2006). In addition, lower PC concentrations led to decreased PtdCho concentrations. Phosphatidylcholine is the major phospholipid component of cell membranes and is necessary for the structure and function of all cells and is crucial for sustaining life (Kent, 1995). The observation that the concentration of PtdCho decreased with advancing apoptosis stages has been reported earlier (Engelmann *et al*, 1996; Glunde *et al*, 2002).

The increase of CH_2_ and CH_3_ resonances of free-fatty acid chains, as well as the accumulation of PUFAs, represents two other characteristics associated with ongoing cell death processes (Blankenberg *et al*, 1997; Griffin *et al*, 2003). This change in the degree of unsaturation of fatty acid chains, together with the detected neutral lipid accumulation and the observation of a significant drop in PtdCho, could result from either activated PtdCho catabolism or inactivated PtdCho anabolism. Considering the PtdCho catabolic pathway, two options are possible. The first option involves the activity of phospholipase A2 to produce lyso-PtdCho (Hakumaki *et al*, 1999), which in turn can be hydrolysed into free-fatty acids and GPC. In our experimental setup, we did not observe an increase in GPC, making this catabolic pathway more unlikely. The second catabolism pathway involves the action of PtdCho-specific phospholipase C. In our system, this is also not very likely as the reaction product, PC, does not increase, but drops after imatinib treatment. Considering the PtdCho anabolic pathway, as the cells are preparing to die, they could be reducing their uptake of choline; hence, less PtdCho would be synthesised. This would lead to an accumulation of DAGs, which could then be converted to TAGs to prevent the disruption of cellular metabolism. This is most likely the cause for the accumulation of TAGs in our cells and the most likely explanation for the decrease in PtdCho and accumulation of neutral lipids and fatty acids. The accumulated fatty acids can enter the mitochondrial or peroxisomal *β*-oxidation pathway, in which they are broken down to acetyl-CoA or acetate. The overall concentration of acetate, the end product of peroxisomal *β*-oxidation, was not significantly changed upon imatinib treatment. However, it is possible that the lipid synthesis and cycling may have allowed for peroxisomal *β*-oxidation and energy production to replace mitochondrial *β*-oxidation during imatinib exposure.

The generally accepted action of *ABCB1* (*MDR1*) is to reduce intracellular drug accumulation through p-glycoprotein-mediated efflux, thus reducing drug concentrations at the intracellular effector sites. K562 cells, however, did not reveal any changes in p-glycoprotein expression. Increased intracellular concentrations of imatinib can be explained by a dose-escalating regimen as well as by membrane changes detected by NMR. Studies have shown that apoptosis, as detected in our imatinib-treated cells, leads to changes in membrane fluidity and porosity, which in turn go parallel with a reduction in p-glycoprotein function (Ferte, 2000; Kok *et al*, 2000).

In summary, we studied the time-dependent effects of imatinib on human Bcr-Abl^+^ K562 cells over an observation period of 4 weeks. Initially, we observed a shift from energy production by anaerobic glycolysis to mainly mitochondrial oxidation. Later metabolite changes reflected apoptosis and, at an even later stage, necrosis. Each of these imatinib-induced changes resulted in characteristic intracellular metabolite patterns (Figure 5) and showed good correlation with molecular changes such as protein expression. Therefore, the use of multi-NMR spectroscopy in cell extracts can help to evaluate the mode of action of anticancer drugs on the molecular level. On the basis of our results, it seems reasonable to assess in future studies to which extent these results can be translated into diagnostic tools for monitoring imatinib therapy.

## Figures and Tables

**Table 1A:** Absolute intracellular concentrations (nmol g^−1^ cell weight) of ^13^C-glucose and its intermediates (lactate and TCA cycle intermediates), high energy phosphates (PCr, NTP and NDP), NAD^+^ (*μ*mol l^−1^ per 10^7^ cells), phospholipids (phosphatidylcholine and phosphocholine), acetate and polyunsaturated fatty acids (PUFA) and their ratios (NTP/NDP and PME/PDE) calculated from K562 cell extracts as assessed by ^1^H-, ^13^C- and ^31^P-NMR

**A**	**Control**	**0.1 *μ*M 1 week**	**0.2 *μ*M 2 weeks**	**0.4 *μ*M 4 weeks**
^13^C-lactate	1041.2±376.6	639.1±145.4^*^	153.8±62.3^*^	210.1±36.7^**^
TCA cycle intermediates	313.2±69.5	448.4±93.3^*^	299.1±41.1	143.8±66.3^**^
Glucose intracell	69.7±13.9	204.1±76.4^*^	192.9±43.2^*^	213.0±85.6^**^
*(NTP/NDP)*	*6.67±1.50*	*9.42±0.94* ^*^	*6.66±0.96*	*1.75±0.60* ^**^
NAD^+^	1.32±0.46	0.74±0.30^**^	0.54±0.27^***^	0.41±0.22^***^
PCr	540.2±247.1	230.2±50.2^*^	270.1±9.32^***^	ND
PC	1448.3±205.2	1086.4±53.4^*^	765.1±82.0^***^	737.5±35.4^***^
PtdCho	2351.3±432.3	2052.1±411.2	1794.3±293.3^*^	118.6±225.3^**^
*(PME/PDE)*	*4.31±1.52*	*4.01±1.01*	*2.67±0.13* ^*^	*2.07±0.56* ^*^
Acetate	413.1±81.8	368.7±149.5	668.9±247.2	411.0±181.2
PUFA	2512.3±264.3	3094.2±445.3	3185.3±293.4^*^	4784.7±227.5^***^

Abbreviations: NAD^+^=nicotineamide adenine dinucleotide; ND=not detectable; NDP=nucleoside diphosphate; NTP=nucleoside triphosphate; PC=phosphocholine; PCr=phosphocreatine; PDE=phosphodiester; PME=phosphomonoester; PtdCho=phosphatidylcholine; PUFA=polyunsaturated fatty acids; TCA=tricarboxylic acid.

Values are presented as means±s.d. of 3–5 independent experiments (control and 1-week experiments were performed with *n*=5; 2- and 4-week experiments with *n*=3).

Significance levels: ^*^P<0.05; ^**^P<0.005; ^***^P<0.001 were determined by ANOVA (with *post hoc* pairwise multiple comparison Tukey-test).

**Table 1B:** Absolute concentrations of ^13^C-labelled extracellular glucose and lactate (mmol l^−1^ per 10^7^cells) in K562 cell media as calculated from ^1^H-NMR spectra

**B**	**Control**	**0.1 *μ*M 24 h**	**0.1 *μ*M 48 h**	**0.1 *μ*M 96 h**	**0.2 *μ*M 2 weeks**	**0.4 *μ*M 4 weeks**
Glucose extracell	2.23±0.19	2.39±0.08	2.89±0.12^**^	3.80±0.15^***^	3.91±0.26^***^	4.53±0.76^***^
Lactate extracell	0.303±0.033	0.185±0.019^**^	0.106±0.004^***^	0.037±0.005^***^	0.030±0.001^***^	0.021±0.013^***^

Results are means±s.d. (all groups *n*=5).

Significance levels: ^**^P<0.005; ^***^P<0.001 were determined by ANOVA (with *post hoc* pairwise multiple comparison Tukey-test).

**Table 2:** Intracellular imatinib concentrations in K562 as calculated based on HPLC-MS/MS data

	**24 h**	**48 h**	**72 h**	**96 h**	**2 weeks**	**4 weeks**
Imatinib dose	0.1 *μ*M	0.1 *μ*M	0.1 *μ*M	0.1 *μ*M	0.2 *μ*M	0.4 *μ*M
Imatinib intracell	0.015±0.003	0.015±0.001	0.019±0.005	0.018±0.002^*^	0.035±0.005^**^	0.065±0.009^***^

The values were calculated as *μ*M imatinib per 10^7^ cells and represent means±s.d. of four independent experiments.

Statistical significance: ^*^P<0.05; ^**^P<0.005; ^***^P<0.001 when values were compared with the 24-h value(s). Comparison by ANOVA indicated statistically significant differences with *P*<0.001.
